# Immune Surveillance of Senescent Cells

**DOI:** 10.1093/geroni/igab046.952

**Published:** 2021-12-17

**Authors:** Scott Lowe

**Affiliations:** Memorial Sloan Kettering Cancer Center & Howard Hughes Medical Institute, New York, New York, United States

## Abstract

Cellular senescence involves a stable cell cycle arrest and a secretory program that modulates the tissue environment. In cancer, senescence acts as a potent barrier to tumorigenesis and, though many cancers evade senescence during the course of tumor evolution, ionizing radiation and conventional chemotherapy can, to varying degrees, induce senescence in tumor cells leading to potent anticancer effects. Conversely, the aberrant accumulation of senescent cells can reduce regenerative capacity and lead to tissue decline, contributing to tissue pathologies associated with age or the debilitating side-effects of cancer therapy. Our laboratory studies mechanisms of cellular senescence with the ultimate goal of developing strategies to modulate senescence for therapeutic benefit. We have focused on how senescent cells trigger immune surveillance to facilitate their own elimination or, when that fails, how synthetic immune cells (i.e. CAR T cells) can be directed to eliminate senescent cells. Recent advances in understanding senescent cell surveillance by the immune system will be discussed.

